# A Light-Powered Micropump with Dynamic Collective Behavior for Reparation

**DOI:** 10.3390/nano14060517

**Published:** 2024-03-14

**Authors:** Yunyu Sun, Hao Wang, Jiwei Jiang, Hui Zhang, Limei Liu, Keying Zhang, Bo Song, Bin Dong

**Affiliations:** 1 Institute of Functional Nano & Soft Materials (FUNSOM), Jiangsu Key Laboratory for Carbon-Based Functional Materials & Devices, Soochow University, Suzhou 215123, China; sunyunyu2008@ahszu.edu.cn (Y.S.); hwang415@stu.suda.edu.cn (H.W.); huizhang@suda.edu.cn (H.Z.); 2 School of Electronic Science & Engineering, Southeast University, Nanjing 210096, China; 3 Anhui Key Laboratory of Spin Electron and Nanomaterials of Anhui Higher Education Institutes, School of Chemistry and Chemical Engineering, Suzhou University, Suzhou 234000, China; szxyzky2009@ahszu.edu.cn; 4 College of Mechanical Engineering, Yangzhou University, Yangzhou 225127, China; limeiliu@yzu.edu.cn; 5 Laboratory of Advanced Optoelectronic Materials, College of Chemistry, Chemical Engineering and Materials Science, Soochow University, Suzhou 215123, China; songbo@suda.edu.cn

**Keywords:** micropump, light-driven, collective behavior, reparation

## Abstract

Inspired by the collective behaviors of active systems in nature, the collective behavior of micromotors has attracted more and more attention in recent years. However, little attention has been paid to the collective behavior of the immobilized micromotor, i.e., the micropump. In this paper, a unique pentacene-based micropump is reported, which demonstrates dynamic collective behavior activated by white light irradiation. The light irradiation may generate the photochemical reactions between pentacene and water, leading to the electroosmotic flow. As a result, this micropump is capable of pumping the surrounding solution inward along the substrate surface based on the electroosmosis mechanism. Intriguingly, the inward pumping causes the agglomeration of the tracer particles on the surface of the micropump. In addition, the aggregation can migrate following the change in the light irradiation position between two adjacent micropumps. Based on the aggregating and migrating behaviors of this pentacene-based micropump, we have achieved the conductivity restoration of the cracked circuit.

## 1. Introduction

In the past decades, micromotors have attracted more and more attention and arecapable of moving autonomously in a liquid environment in the presence of external stimuli or chemical fuels. In order to realize the moving behavior, the asymmetric structure of micromotors or the asymmetric distribution of the energy around micromotors has been adopted by introducing functional materials into the structure or applying external stimuli from one side [1,2,3]. To date, several mechanisms have been established to clarify the self-propulsion behavior, such as bubble propulsion [4,5], self-diffusiophoresis [6,7], surface tension gradient [8], self-electrophoresis [9,10], etc. Based on the moving property, micromotors are potentially applied in the fields of sensing [11,12], microelectronics [13], precise medicine [14,15], environmental remediation [16,17], cargo delivery [18,19], etc. Compared with toxic chemical fuels (hydrogen peroxide, hydrazine, Br_2_ or I_2_ solutions, etc.), external fields [20,21,22,23], such as light, ultrasound, magnetic fields and electric fields, have also been utilized to physically stimulate the motion of a micromotor [24,25,26]. Inspired by the schooling behavior of living organisms, the collective behavior of micromotors has become one of the research focuses in recent years [27]. In response to external stimuli, micromotors demonstrate different collective behaviors (such as the light-induced “predator–prey” behavior [28], light-activated dynamic assembly of “living crystals” [29], light-navigated migration of micromotors swarm [30] and the formation of ribbon structures activated by the light [31] or magnetic field [32]).

A micropump is often obtained by directly immobilizing the micromotor on the surface of a substrate and shares some common mechanisms with the micromotor, such as electrophoresis [10], diffusiophoresis [33,34,35,36] and surface tension gradient [37,38]. Recently, as an easily obtainable and remotely controllable energy source, light may not only eliminate the utilization of toxic chemical fuels for the micropump but is also capable of pumping the surrounding fluids near the light-responsive pump. When exposed to light, a micropump that consisted of light-responsive materials could induce photo-degradation [39], photocatalytic reaction [40] and photo-thermal conversion [41] to generate the appearance of solutal buoyancy, diffusiophoresis, electroosmosis and thermal buoyancy, which resulted in the pumping behavior of the fluid and tracer particles. Similarly, collective behavior in a micropump system has also been reported [39,42,43]. For instance, positive tracer particles move toward the Si/Pt micropump and aggregate on its surface [40]. Despite this progress, the collective behavior observed in the micropump system still lacks dynamic features, thus hindering its potential in different applications.

For electronic devices, cracks in the conductive pathway may occur, which can be caused by operational fatigue or accidental cutting or scratching. Due to the increasing complexity of the electrical circuit, even a microscopic crack may lead to the failure of the whole device. The restoration of the electrical conductivity after the circuit breakdown is, thus, of great importance. An electrical device with healing capability offers a potentially promising solution to this challenge. Healable electrical conductors can be realized by autonomous methods or through non-autonomous means. To date, several strategies have been developed to achieve repair of the conductivity failure. Among others, the microcapsule/pipeline-based method relies on the encapsulation of the microcapsule containing the conductive materials [44,45], such as a carbon nanotube, liquid metal, etc., which can be released at the cracked area upon capsule rupture. The microcapsule/pipeline-based system has several limitations: it cannot heal the damage at the same location multiple times and it cannot heal macroscopic cracks. On the other hand, the conductivity restoration can also be realized through the utilization of the intrinsic properties. For example, composites containing supermolecular (hydrogen bonding) polymers and metallic microparticles or metal ions have been demonstrated to be electrically healable with the aid of the manual pressing of the broken area [46,47]. Two-layered composites consisting of surface conductive materials and underlying healable polymers could also heal them based on the Diels–Alder reaction or water treatment [48], etc. Although the intrinsically self-healing system can efficiently recover the electrical conductivity, the stimuli applied to trigger the healing process, such as high temperature or mechanical stress, may cause additional damage to the electronic devices. The intrinsically self-healing system not only requires a long recovery time but also is not compatible with the printing process and depends on special chemistries to start the restoration, which could be inhibited by the ambient condition. Additionally, the conductivity restoration could also be realized by directly applying electrical healing agents, such as TTF-TCNQ charge transfer salt [49], a carbon nanotube [50] or functionalized carbon black [51], etc. to the cracked area. However, this method is not ideal for repairing microscale breaks because it cannot realize the localized delivery of the healing agents without influencing the nearby functional parts. Therefore, it is still challenging to develop a conductivity restoration system that can realize the precise delivery of the healing agent to the specific defect site in a repeatable and non-invasive fashion.

In this paper, we report a pentacene-based micropump with dynamic collective behavior (aggregating and migrating behavior), which may find a potential application in conductivity restoration. When immersed in deionized water, protons are generated through the photochemical reactions between the pentacene and water under light illumination. The fast diffusion of protons may generate an electric field, which causes the water flow to move toward the micropump along the substrate based on the electroosmosis mechanism. As a consequence, accompanied by the water flow, the tracer particles near the substrate are also pumped toward the pentacene micropump. Interestingly, the pumping direction is irrelevant to the surface charges of the tracer particles. Furthermore, the tracer particles could aggregate near the irradiation center on the pentacene surface. More interestingly, the aggregating behavior is dynamic. The aggregates move as the irradiation spot changes. Based on this dynamic aggregating behavior, we have realized the conductivity restoration of a cracked circuit.

## 2. Results and Discussion

**Figure 1:**
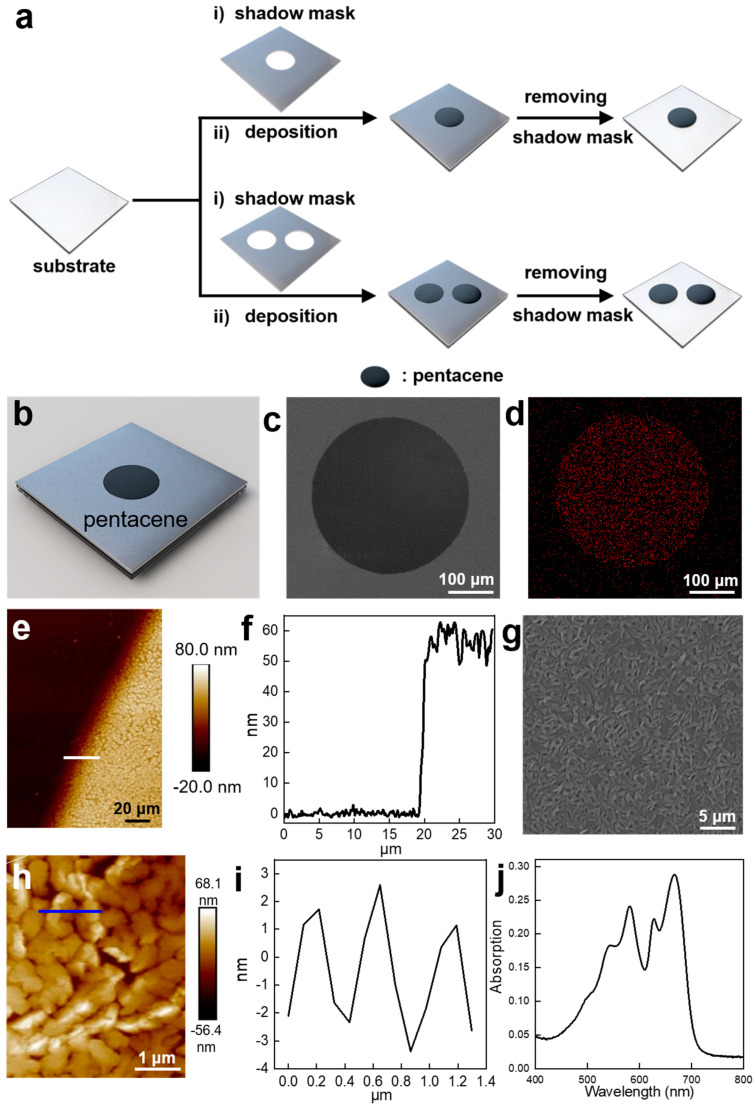
Fabrication and characterization of the pentacene-based micropump. (**a**) Schematic illustration showing the fabricating process of the micropump based on pentacene. (**b**) Schematic illustration and (**c**) scanning electron microscope (SEM) image of the micropump. (**d**) The EDX analysis of the micropump for carbon element. (**e**) Atomic force microscopy (AFM) image and (**f**) the section analysis showing the surface morphology and the thickness of the micropump. (**g**) Enlarged SEM image of the micropump. (**h**) Enlarged AFM image of the micropump. (**i**) The section analysis of the surface shown in (**g**). (**j**) UV–Vis–NIR spectrum of the pentacene.

The fabricating process of the micropump is illustrated in Figure S1a in the Supporting Information. The pentacene is directly deposited on the surface of a substrate (glass or silicon) by utilizing the shadow mask evaporation method. The micropump has a disc-like shape with a diameter of approximately 430 μm (Figure 1b,c). The EDX result demonstrates that the carbon element distributes homogeneously throughout the whole microstructure (Figure 1d), confirming the successful deposition of pentacene. In addition, as can be seen from the Atomic Force Microscope (AFM) image shown in Figure 1e, the surface of the disc-like micropattern is rough and the current micropattern has a thickness of around 55 nm (Figure 1f) [52]. We further studied the detailed structure of this micropump. The micropump is composed of many small pentacene microcrystals, as can be visualized from the enlarged SEM (Figure 1g) and AFM images (Figure 1h). Through the AFM section analysis (Figure 1i), the surface roughness can, thus, be determined to be around 5 nm, which offers more sites for absorbing light energy. The pentacene micropump shows a broad absorption band in the visible range (Figure 1j), indicating that pentacene may be able to efficiently absorb white light, thus facilitating the following photocatalytic reaction.

**Figure 2:**
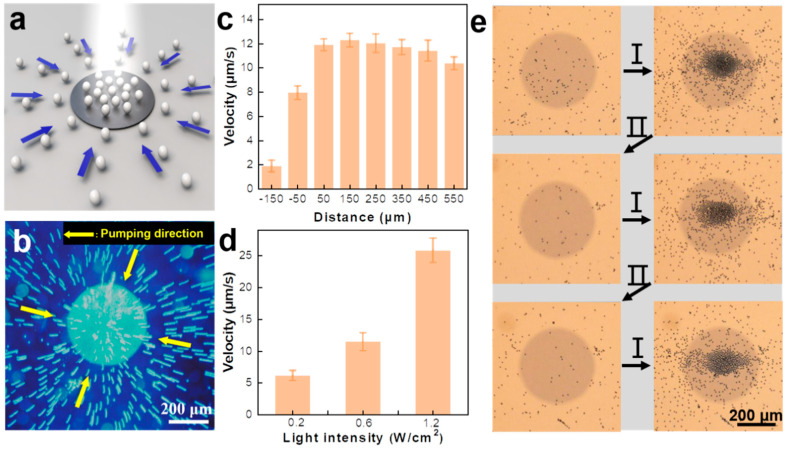
(**a**) Schematic illustration and (**b**) overlaid optical microscopic image showing the pumping behavior of the micropump. White balls: tracer particles. Blue arrows: the direction of pumping behavior. (**c**) The average velocity of tracer particles as a function of the distance from the border of the pentacene micropump. Light intensity: 0.6 W/cm^2^. (**d**) The average velocity of tracer particles under different light intensities. (**e**) The aggregating–dispersing recycles of tracers based on the same micropump. I and II represent the aggregation and dispersion states, respectively. Light intensity: 1.2 W/cm^2^.

To study the pumping behavior of the resulting pump, carboxylate-functionalized polystyrene microspheres (PS-COOH MPs) are used as the tracer particles. Prior to the white light irradiation, the PS-COOH MPs around the micropump remain motionless, indicating the absence of the pumping behavior. Surprisingly, under white light irradiation (light intensity: 0.6 W/cm^2^), the PS-COOH MPs start moving toward the micropump and aggregate near the center of the irradiation spot, which is located on the surface of the micropump, indicating that the white light irradiation activates the pumping process of the micropump (Figure 2a,b and Video S1 in the Supporting Information). The average velocity of the tracer particles increases as they move closer to the micropump and reaches a maximum (12 μm/s) when they move to a position 300 μm away from the micropump boundary and then remains almost unchanged (Figure 2c). The velocity of the tracer particles is also strongly influenced by the light intensity. The pumping velocity increases proportionately with the increasing irradiation intensity (Figure 2d). The micropump can reach a pumping speed of 25.6 μm/s under 1.2 W/cm^2^ light irradiation. Upon the removal of the irradiation light, the pumping behavior of the pentacene microstructure stops almost immediately. In addition, the light-sensitive pumping behavior of the pentacene microstructure can be repeated many times. As illustrated in Figure 2e, after three aggregation–dispersion cycles, the aggregation of the tracer particles caused by the pumping behavior can be well observed.

**Figure 3:**
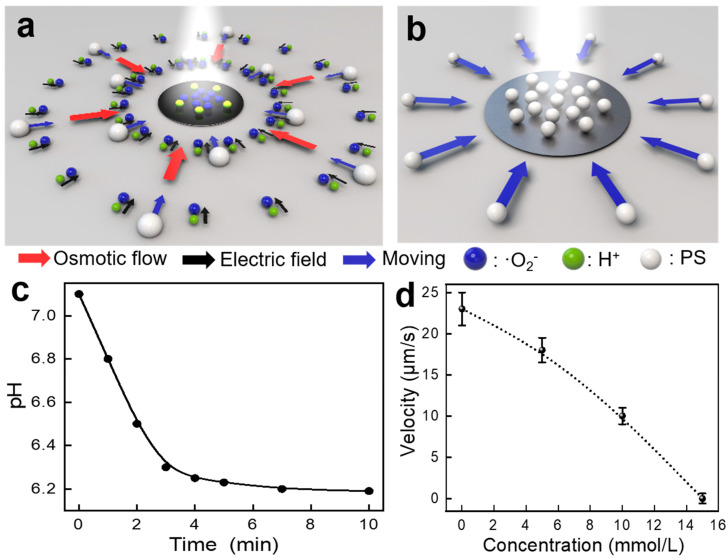
(**a**,**b**) Schematic illustration of mechanism of the light-activated pumping behavior. (**c**) The pH of the solution changes with the illuminating time (light intensity 1.2 W/cm^2^). (**d**) The velocity of the tracer particles decreases as the concentration of NaCl solution increases.

Pentacene is a semiconductor. Based on the above findings and other light-driven systems [10,53], a possible mechanism is proposed. Under white light irradiation, the pentacene would generate a photocatalytic reaction to create a photogenerated electron/hole pair. Then, the photochemical reaction between the water and the hole is induced, which generates H^+^ ions. As shown in Figure 3c, the pH decreasing with time indicates the generation of protons during the light irradiation process. The generation of protons under white light irradiation is further confirmed by the pH indicator (pyranine). As shown in Figure S1a–d, the fluorescent intensity of pyranine depends on the solution pH, which could be utilized to detect the pH variation in the solution. Then, after dropping the pyranine solution into the solution with the micropump, white light irradiation is applied. As shown in Figure S2e–h, the overall fluorescence gradually fades with the increasing irradiation time, further indicating the generation of protons.

Due to the different mobilities of the different ions, they diffuse at different speeds, and H^+^ ions move the fastest. As a result, an electrical field is generated, and it directs from the outside to the pentacene. Because the absolute value of ξ potential of the glass slide surface is negative, the double layer near the glass surface is positive. Consequently, the corresponding electroosmotic fluid flow moves from the outside toward the pentacene micropattern, leading to the motion of the tracer particles inward (Figure 3a,b). The concentration of ions increases close to the micropump, generating a stronger force of electroosmotic flow, which results in a higher average velocity. In addition, in a control experiment, we place the pentacene micropattern in water containing different concentrations of NaCl. The pumping speed decreases as the concentration of the NaCl solution increases. No pumping behavior of tracers near the glass surface is found when the concentration of the NaCl solution is 15 mmol/L (Figure 3d and Video S2 in the Supporting Information). Thus, when the concentration of ions (Na^+^ and Cl^−^) in the solution is high enough, which is much higher than the concentration of ions (H^+^) produced by the micropump during white light irradiation, the high concentration of ions in the solution disrupts the electric field, resulting in the inability to generate electroosmotic flow and, thus, the inability to actuate the particles. Furthermore, since the movement of the tracer particles is due to the flow of liquid resulting from the electroosmotic flow, the direction of the pumping behavior should be the same as the direction of the electroosmotic flow. As evidenced by the fact that the corresponding movies for the positive and neutral polystyrene microspheres are shown in the Supporting Information as Video S3, the direction of the pumping behavior is always inward, which indicates that the pumping behavior is irrelevant to the surface charges of the tracer particles, which further indicates the mechanism of the electroosmotic fluid. The average velocity is associated with the electrical properties of the tracer particles, and the positive tracer particles move fastest because the electrical field directs them from the outside to the pentacene, which further demonstrates that the electroosmotic flow plays a more significant role than the electrophoretic force (Figure S2).

**Figure 4:**
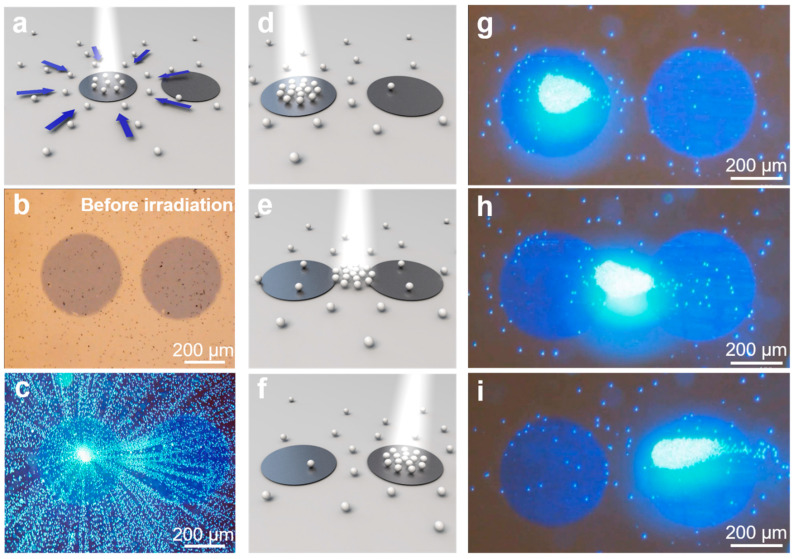
Schematic (**a**) and overlaid image (**b**,**c**) showing the aggregating behaviors of tracer particles on the focus spot in a system consisting of two adjacent pentacene microstructures. (**d**–**f**) Schematic showing dynamic migration of the aggregation tuned by the light. (**g**–**i**) The corresponding optical microscopic images of migrating process. Light intensity: 1.2 W/cm^2^.

In addition, we discovered that the moving and aggregating behaviors of tracer particles can be realized between two adjacent pentacene microstructures. As shown in Figure 4a–c and Video S4 in the Supporting Information, the light induces the aggregation of the tracer particles near the focus spot of the irradiation on the left micropump. Then, the aggregation of the tracer particles can be guided when the white light focus center of the illumination changes to a different location. When the irradiation center moves to the right micropump, the aggregation of tracer particles moves together toward the new irradiation center (Figure 4d–i and Video S5 in the Supporting Information). This phenomenon is likely due to the fact that the tracer particles always aggregate near the illumination center. When the irradiation focus changes to a new position, the aggregation moves again following the illumination spot, and all the tracer particles re-aggregate near the new irradiation center. This result indicates that the aggregative behavior of the tracer particles induced by the micropump is quite dynamic, and the dynamic aggregation/re-aggregation process can be simply triggered by the position of the applied light irradiation. This is also in contrast to the previously reported aggregative behavior, which cannot realize the precisely controlled and dynamic movement of the aggregation [28,54].

**Figure 5:**
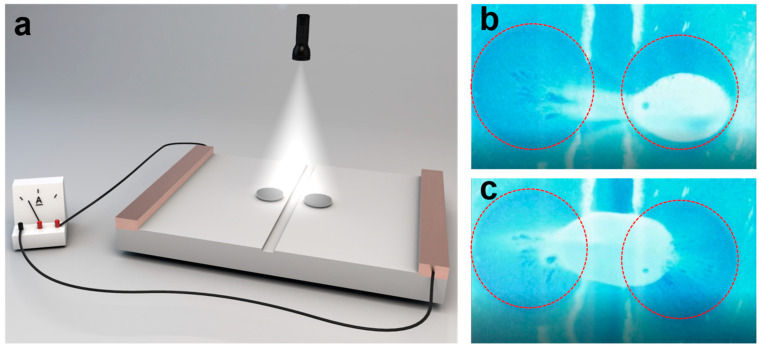
(**a**) Schematic showing the application in light-controlled reparation of the cracked conductive path. (**b**,**c**) The optical microscopic images obtained from Video S6 in the Supporting Information showing the dynamic repairing process. The red circles are the micropumps.

Since the current micropump could induce the aggregative behavior at a targeted position that is controlled by the location of the light irradiation, we applied this observation in the field of the reparation of the broken circuit (Figure 5a). As a proof-of-concept, we deposited thin pentacene films on ITO glass and then immersed them into a solution containing gold nanoparticles. The gold nanoparticles with a diameter of around 500 nm act as the repairing agent in the solution. The white light irradiation can still induce the aggregation behavior of the gold nanoparticles near the irradiation spot (Figure 5b). Under white light irradiation, the gold nanoparticles move toward the illuminated area with a velocity of 29.2 μm/s, which is similar to that of the above findings, and they form aggregates near the light spots. Once the light is withdrawn, the movement stops, indicating the aggregation is dynamic. The original ITO is conductive with a resistance of approximately 25.8 ohms. Then, we create a crack on the surface of the ITO substrate by wet etching. After cracking, the resistance of the film increases dramatically to around 1 × 10^7^ ohm and the film is not conductive. When the white light irradiation is applied to the surface of the cracked surface of the pentacene/ITO, the gold nanoparticles aggregate toward the illumination spot, resulting in the coverage of the gold nanoparticles on the surface of the cracked area (Figure 5c and Video S6 in the Supporting Information). The resistance of the ITO with the crack repaired by gold nanoparticles is around 75 ohms, which is comparable to the original value and far below that of the cracked ITO, indicating the successful reparation of the cracked area.

## 3. Conclusions

In conclusion, we report a pentacene-based micropump. Because of the photocatalytic reaction of the pentacene semiconductor, the pentacene-based micropump could pump the surrounding solution based on the electroosmotic mechanism under white light irradiation. In addition, the tracer particles exhibit an inward pumping behavior near the substrate, causing the aggregation of the tracer particles on the pentacene microstructure. Moreover, the pumping and aggregation behavior are quite dynamic and are controlled by the on–off and the irradiation location of the applied light. Additionally, the light-controlled aggregation in the current study may have great potential in the field of crack reparation of a broken circuit.

## 4. Experimental Procedure

*Materials:* The pentacene was obtained from Tokyo Chemical Industry Co., Ltd. (Tokyo, Japan) Gold spherical powder (diameter 0.5–0.8 μm) was purchased from Alfa Aesar. Tracer particles with different surface charges, i.e., the positively charged PS particle (diameter 6–6.9 μm), the negatively charged PS particle (diameter 8–8.9 μm) and the neutral PS particle (diameter 7–7.9 μm) were obtained from the Aladdin company (Bay City, MI, USA). The ITO glass was obtained from Zhuhai Kaivo Optoelectronic Technology Co., Ltd. (Zhuhai, China).

*Fabrication:* The pentacene was directly deposited onto a variety of substrates, such as glass, silicon and ITO glass by utilizing the thermal evaporation technique with a thickness of approximately 55 nm, as determined by the quartz crystal microbalance in the presence of a shadow mask. Thermal evaporation was performed on a high vacuum system (Kurt J. Lesker, working pressure < 2 × 10^−4^ Pa). The deposition rate was about 0.015 nm/s, and the substrate temperature was kept at room temperature.

*Characterizations:* SEM measurements were performed on a Carl Zeiss Supra 55 scanning electron microscope (Jena, Germany) with an elemental analysis (EDX) attachment. For the SEM examination, a thin layer of pentacene was deposited on a silica substrate prior to the SEM observation. AFM measurement was carried out on a Bruker Dimension Scanning Probe Microscope (Billerica, MA, USA). The optical microscopic image was obtained by utilizing a Leica DM4000M fluorescence microscope (Wetzlar, Germany). UV–Vis–NIR spectra were recorded using a PerkinElmer Lambda 750 UV-Vis-NIR spectrophotometer (Waltham, MA, USA).

*The micropump experiments:* For the micropump experiments, the pentacene micropattern was immersed in a 200 μL solution containing tracer particles. The micropump was then actuated by the application of light irradiation (X-cite 120Q, Excelitas Technologies, Waltham, MA, USA). The light irradiation intensity in the current study was around 1.2 W/cm^2^ unless otherwise mentioned. The pumping behavior of the micropump was then observed and recorded under an OLYMPUS BX51 optical microscope (Tokyo, Japan). The captured video could then be utilized to analyze and obtain the pumping velocity of the resulting micropump by utilizing PhysVisPhysmo v2.0 software. The speed of tracer particles was analyzed using MATLAB R2021 software. In the pumping behavior observation experiment, we placed the micropump in water and subsequently added tracer particles. It was left for a period of time (20 min) to ensure that all the tracer particles completely sunk to the bottom. Subsequently, the center of the white light irradiation was aimed at the micropump, and the movement of the tracer particles was observed. While observing the pumping behavior in the two adjacent pentacene microstructure systems, the silicon chip with two pentacene microstructures was placed in the water with the tracer particles. The white light irradiation was first applied to the micropump on the left, and after the particles had gathered, we moved the irradiation center of light to the micropump on the right. The above back-and-forth motion experiment was repeated three times.

*The reparation of the cracked conductive path:* For the influence of the voltage section, two voltages, V1 and V2, were applied, and V1 was set to 1.5 V, while V2 was changed from 0 to 1.3 V. For the self-healing experiment, a crack was then obtained by the wet etching method. Namely, an etching solution with 1 mol/L FeCl_3_ and 1 mol/L HCl was prepared. We packed ITO glass with polyimide tape and reserved a 200 μm gap without polyimide tape. Then, the ITO glass was immersed in the etching solution. After 20 min, the ITO glass was taken out and washed with DI water. After drying, the polyimide tape was pulled off. Lastly, pentacene micropattern was deposited on both sides of the crack. A droplet of the solution containing the repairing particles, i.e., the 500 nm gold nanoparticles, was then added dropwise onto the pentacene-coated ITO glass with a crack and subjected to light irradiation. After the gold nanoparticles aggregated near the cracked area, the excess solution was removed, and the glass was dried at room temperature. The ITO was then measured by utilizing a DC power station to test the conductivity using a Fluke 15B+ Digital Multimeter (Everett, WA, USA). Lastly, the ITO was connected with a copper wire and connected externally with an ammeter to act as a guide for the conductance and non-conductance of the repaired and non-repaired structures. For the switch experiment, the microdevice was fabricated in a similar fashion to the self-healing process but with no drying.

*The mechanism experiments:* To demonstrate the generation of protons during the light irradiation process, the pH indicator (pyranine) was added to the solution. In order to study the sensitivity of the proton indicator, we prepared 0.1 mmol/L pyranine solutions with different pH levels (6.8, 6.4 and 6.0). The fluorescence images were then observed and recorded under an OLYMPUS BX51 optical microscope. After dropping 200 μL pyranine solution (0.1 mmol/L) into the solution with micropump, the fluorescence of the solution was also observed and recorded under an OLYMPUS BX51 optical microscope. The intensity of the fluorescence was analyzed using ImageJ 2.1.0 software. The change in the pH of the solution with the micropump during the white light irradiation was determined using a commercial pH meter. Firstly, the water with the micropump was measured at different time points (per 2 min) under the white light irradiation. Then, a commercial pH meter was used to determine the pH at each time point.

*The pumping behavior of the micropump in the NaCl solution:* To analyze the pumping behavior of the micropump in the NaCl solution, the micropump was immersed in NaCl solutions of different concentrations. The pumping behavior was recorded using an OLYMPUS BX51 optical microscope, and then the speed of the tracer particles was analyzed.

## Data Availability

Data are contained within the article and Supplementary Materials.

## Supplementary Materials

The following supporting information can be downloaded at https://www.mdpi.com/article/10.3390/nano14060517/s1, Figure S1. (a–d) The fluorescence images and intensity of the pH indicator with different pH. (e–h) The change of fluorescence images of the solution with micropump during white light irradiation; Figure S2. The velocity of the tracer particles with different electricity.; Video S1. The pumping behavior of the pentacene micropump driven by light. Light intensity 0.6 W/cm^2^. 20 × speed; Video S2. The pumping behavior of the trace particles in the NaCl solution. Light intensity 1.2 W/cm^2^. 10 × speed; Video S3. The pumping direction of the trace particles with positive or neutral charge. Light intensity 1.2 W/cm^2^. 30 × speed; Video S4. The aggregation of tracer particles on the focus spot in a system consisting of two adjacent pentacene micropumps. Light intensity 1.2 W/cm^2^. 50 × speed; Video S5. The dynamic movement of the aggregation tuned by the light. Light intensity 1.2 W/cm^2^. 50 × speed; Video S6. The application of pumping in crack reparation based on pentacene micropump. Light intensity 1.2 W/cm^2^. 10 × speed.
