# Supplementary material for: A Light-Powered Micropump with Dynamic Collective Behavior for Reparation
Source: Nanomaterials (Basel). 2024 Mar 14;14(6):517. doi: 10.3390/nano14060517 (PMC10975955; doi:10.3390/nano14060517)
Supplement: Supplementary file 1 [file nanomaterials-14-00517-s001.zip › supporting information.pdf]

Supporting information

# A Light-Powered Micropump with Dynamic Collective Behavior for Reparation

Yunyu Sun[1, 2, 3, #], Hao Wang[1, #], Jiwei Jiang[1, *], Hui Zhang[1], Limei Liu[4], Keying Zhang[3], Bo Song[5] and Bin Dong[1, *]

[1] Institute of Functional Nano & Soft Materials (FUNSOM), Jiangsu Key Laboratory for Carbon-Based Functional Materials & Devices, Soochow University, Suzhou 215123, China; sunyunyu2008@ahszu.edu.cn (Y.S.); hwang415@stu.suda.edu.cn (H.W.); jwjiang@suda.edu.cn (J.J.); huizhang@suda.edu.cn (H.Z.); bdong@suda.edu.cn (B.D.)

[2] School of Electronic Science & Engineering, Southeast University, Nanjing, 210096, China

[3] Anhui Key Laboratory of Spin Electron and Nanomaterials of Anhui Higher Education Institutes, School of Chemistry and Chemical Engineering, Suzhou University, Suzhou 234000, China; szxyzky2009@ahszu.edu.cn.

[4] College of Mechanical Engineering, Yangzhou University, Yangzhou 225127, China; limeiliu@yzu.edu.cn (L.L.)

[5] Laboratory of Advanced Optoelectronic Materials, College of Chemistry, Chemical Engineering and Materials Science, Soochow University, Suzhou 215123, China; songbo@suda.edu.cn

**Video S1.** The pumping behavior of the pentacene micropump driven by light. Light intensity 0.6 W/cm$^2$. 20 × speed.

**Video S2.** The pumping behavior of the trace particles in the NaCl solution. Light intensity 1.2 W/cm$^2$. 10 × speed.

**Video S3.** The pumping direction of the trace particles with positive or neutral charge. Light intensity 1.2 W/cm$^2$. 30 × speed.

**Video S4.** The aggregation of tracer particles on the focus spot in a system consisting of two adjacent pentacene micropumps. Light intensity 1.2 W/cm$^2$. 50 × speed.

**Video S5.** The dynamic movement of the aggregation tuned by the light. Light intensity 1.2 W/cm$^2$. 50 × speed.

**Video S6.** The application of pumping in crack reparation based on pentacene micropump. Light intensity 1.2 W/cm$^2$. 10 × speed.

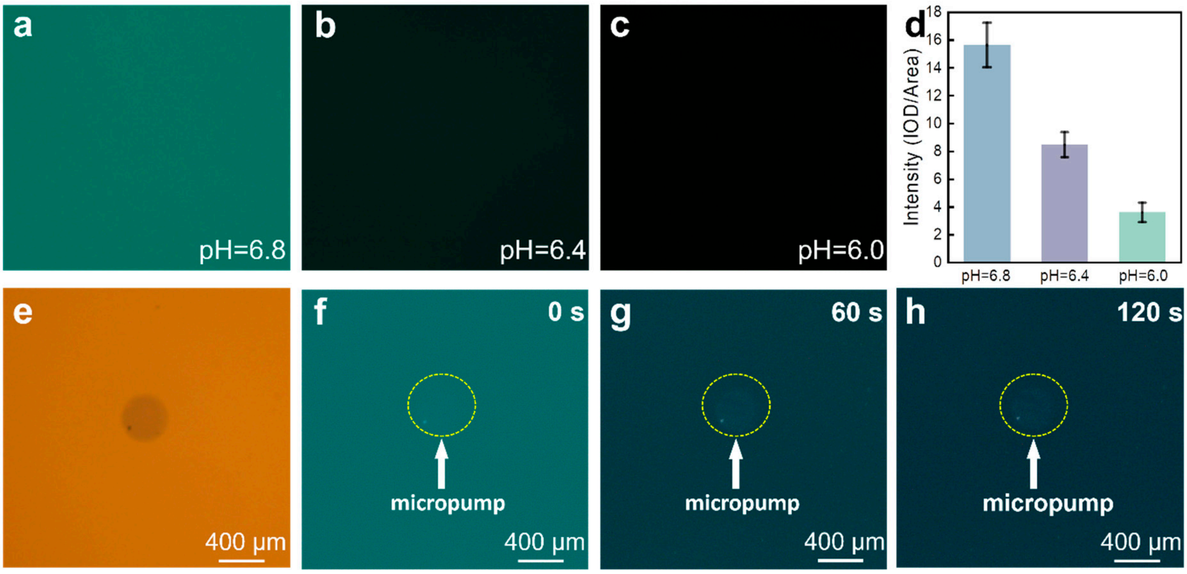


**Figure S1.** (a-d) The fluorescence images and intensity of the pH indicator with different pH. (e-h)

The change of fluorescence images of the solution with micropump during white light irradiation.

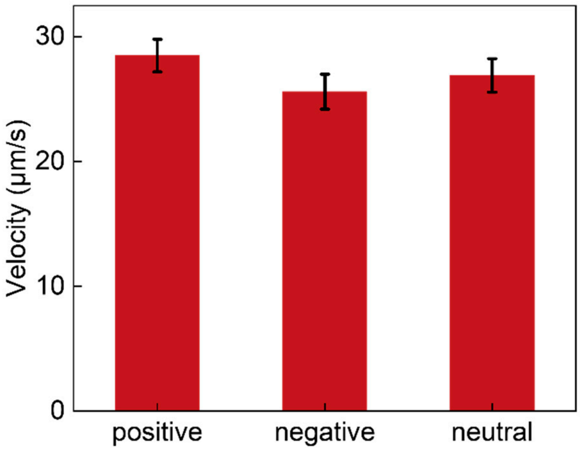


**Figure S2.** The velocity of the tracer particles with different electricity.
